# Horse Grimace Scale Does Not Detect Pain in Horses with Equine Gastric Ulcer Syndrome

**DOI:** 10.3390/ani13101623

**Published:** 2023-05-12

**Authors:** Gustavo Ferlini Agne, Bridget Eileen May, Amy Lovett, Olivier Simon, Catherine Steel, Luiz Santos, Laize Guedes do Carmo, Bianca Barbosa, Laís Cristine Werner, Ruan R. Daros, Andrew A. Somogyi, Benjamin Sykes, Samantha Franklin

**Affiliations:** 1 School of Animal and Veterinary Sciences, University of Adelaide, Roseworthy Campus, Adelaide, SA 5371, Australia; 2 School of Veterinary Medicine, Massey University, Palmerston North 4474, New Zealand; 3 Department of Veterinary Clinical Services, Hong Kong Jockey Club, Sha Tin Racecourse, New Territories, Hong Kong; 4 School of Biodiversity, One Health and Veterinary Sciences, The University of Glasgow, Bearsden, Glasgow G12 8QQ, UK; 5 Graduate Program in Animal Science, School of Medicine and Life Sciences, Pontifícia Universidade Católica do Paraná, Curitiba 80215-901, Brazil; 6 Discipline of Pharmacology, Faculty of Health and Medical Sciences, University of Adelaide, Adelaide, SA 5005, Australia

**Keywords:** horse grimace scale, grimace, pain behaviours, pain scale, horse welfare, equine gastric ulcer, squamous gastric disease, glandular gastric disease

## Abstract

**Simple Summary:**

Stomach ulcers (known as equine gastric ulcer syndrome [EGUS]) are a common finding in horses However, the amount of pain horses with EGUS might experience is currently unknown and this knowledge could allow for better clinical decision making and improved horse welfare. The study aim was to assess the use of a pain scale score system, the Horse Grimace Scale (HGS), in horses suffering from EGUS. Facial photographs for HGS evaluation were taken of all horses. Gastroscopy is the gold standard procedure for diagnosis of EGUS, and involves examining the stomach with a video-endoscope. Horses (*n* = 61) were divided into groups based on the presence and severity of EGUS as determined via gastroscopy. Horses with concurrent lameness or other signs of illness were excluded. Comparisons were made of the HGS between horses, with and without gastric lesions. This is the first study investigating the use of the HGS in horses with and without EGUS. The HGS scores were not influenced by the presence or severity of EGUS and no significant differences were noted between groups. Further studies investigating the use of different pain scales in horses with EGUS are needed.

**Abstract:**

Equine gastric ulcer syndrome (EGUS) is a highly prevalent and presumptively painful condition, although the amount of pain horses might experience is currently unknown. The aims of this study were to determine if the Horse Grimace Scale (HGS) could identify pain behaviours in horses with and without EGUS and if severity would be positively associated with the HGS score. Horse grimace scale scores were assessed blindly using facial photographs by seven observers and involved evaluation of 6 facial action units as 0 (not present), 1 (moderately present) and 2 (obviously present). Lameness examination, serum amyloid A (SAA) measurement and gastroscopy evaluation were performed on all horses. Horses (*n* = 61) were divided into two and three groups based on the presence (yes, no) and severity (none, mild, moderate-severe) of EGUS, respectively. Presence of lameness and elevated SAA (≥50 µg/mL) were used as exclusion criteria. Inter-observer reliability was analyzed by intra-class correlation coefficients (ICC). HGS scores between groups were compared using Welch’s and Brown Forsythe tests (*p* < 0.05). Overall, HGS ICC was “excellent” (0.75). No significant differences (*p* = 0.566) were observed in HGS scores between horses with and without gastric lesions (mean, 95% CI; 3.36, 2.76–3.95 and 3, 1.79–4.20, respectively). HGS was not influenced by the presence or severity of EGUS in this current study. Further studies investigating the use of different pain scales in horses with EGUS are needed.

## 1. Introduction

Equine gastric ulcer syndrome (EGUS) is a highly prevalent disease resulting in lesions of the squamous (Equine Squamous Gastric Disease [ESGD]) and/or glandular (Equine Glandular Gastric Disease [EGGD]) mucosa of the stomach [1]. The prevalence of ESGD is highest in racehorses, affecting 80–100% of Thoroughbreds and up to 87% of Standardbred horses in training [2,3,4]. Although lesions within the glandular mucosa occur less frequently, the prevalence of EGGD is reported to be up to 65% in Thoroughbred racehorses and 64% in sport horses [1,5]. Equine gastric ulcer syndrome represents an important welfare issue and cause of economic losses, due to the potential for decreased performance and productivity [1,6]. The condition is associated with either no specific clinical signs or variable changes such as aggressiveness [7], girth sensitivity and overall sensitivity to touch [8], poor performance [2,6] and, less commonly, weight loss [9,10], diarrhoea [11] and abdominal discomfort, indicating that EGUS could potentially cause visceral pain [1].

The assumption that EGUS is a painful condition is based on overall altered performance and behavioural changes in affected horses. In a previous study including 37 horses that were presented for investigation of girth sensitivity, 12 out of 13 horses undergoing gastroscopy were diagnosed with EGUS. In these horses, the increase in girth sensitivity was thought to be related to visceral pain secondary to EGUS [8]. Another study reported a significant decrease in performance in Thoroughbred racehorses with ESGD, and suggested that this may be related to visceral pain [2]. A decrease in dietary intake and lower oxygen consumption, due to a decrease in tidal volume and alveolar ventilation along with shortened stride length, have been associated with poor performance and could potentially relate to gastric pain [6]. Pain-related behavioural changes, such as increased pawing and aggressiveness pre-feeding [12], have been associated with EGUS and could be linked to pain secondary to gastric acid release in contact with diseased areas. Similarly, self-mutilation is a behavioural response to intense or chronic pain from various sources and has been associated with EGUS [13]. Whilst definitive evidence of pain associated with EGUS is lacking, previous studies in human patients indicate that peptic ulcers can be painful and that an increase in pain severity occurs when peptic ulcers are exposed to hydrochloric acid [14]. Furthermore, approximately 50–60% of non-cardiac chest pain in humans presenting to hospital are related to either gastroesophageal reflux disease (a disease process that resembles ESGD) or duodenal ulceration (which has similarities to EGGD) [15,16]. 

Despite the high prevalence of EGUS there is insufficient information as to whether horses suffering from this disease are truly in pain. Whilst the clinical signs imply there is some discomfort or pain experienced, the exact nature or severity of this pain is unknown. Pain is a known welfare issue and the ability to recognize and determine the amount of pain potentially experienced by horses with EGUS would allow for improved gastric health monitoring, and overall improved horse welfare. Assessment of pain in horses mainly relies on subjective measures, such as composite rating scales, with a variety of scales developed to investigate pain in horses with colic [17,18], orthopedic disease [19] and ophthalmic conditions [20]. The grimace scale, which measures pain based on facial expressions, has been used and validated in multiple species including rodents, cats, pigs, lambs and horses [21,22,23,24]. The Horse Grimace Scale (HGS) has been validated in horses experiencing acute pain, such as following routine castration or suffering from acute laminitis, and has also been used to assess chronic pain associated with dental disorders [21,25,26]. To date, no studies have investigated the use of the HGS in horses with EGUS. Moreover, previous studies using HGS to investigate pain associated with other disease processes did not account for the presence of EGUS as a potential contributor to pain. Given the high prevalence of EGUS, it is important to determine the impact that EGUS would have on pain scales such as the HGS. 

The aims of this present study were to determine the differences in HGS scores between horses with and without EGUS, as well as between horses with different levels of EGUS severity. An additional objective was to evaluate the inter-observer reliability between raters with and without previous HGS scoring experience. It was hypothesized that horses with EGUS would have a higher HGS score compared to control horses and that HGS would be positively correlated with gastric lesion severity. Finally, a higher degree of agreement was predicted between observers with previous HGS scoring experience when compared to observers without previous HGS experience. 

## 2. Materials and Methods

### 2.1. Animals

Horses used included a mix of client-owned horses and horses from the university teaching herd. Client-owned horses were offered a baseline lameness assessment as well as a gastroscopy examination free of charge and were either recruited from the University of Adelaide’s Equine Health and Performance Centre client list, social media or through direct communication about the project. 

Eligible horses included adult horses of any age, breed, sex or discipline. All included horses were determined to be free from known concurrent diseases that could elicit pain or a change in HGS scores, as reported by the owner/trainer and based on initial general physical and lameness examinations. Exclusion criteria included horses receiving medical treatment for EGUS or any other medical condition in the 14 days preceding the study. This study was conducted with Animal Ethics Committee approval under the registration number of S-2020-040. 

### 2.2. Horse Grimace Scale Image Acquisition

Serial photographic images of each horse’s face were taken with the use of a smartphone device (iPhone^®^6, Apple, Adelaide, Australia). All images were acquired at the horse’s own environment or at the University of Adelaide Equine Health and Performance Centre. If image acquisition was performed outside the horse’s own environment, a 20 min period of adaptation was allowed prior to any intervention. Before image acquisition, a halter was placed around the horse’s neck and an additional period of 5 min of acclimatization was provided so the horse could acclimatize to the handler’s presence. Images were then taken from the right, left and frontal aspect of each horse’s face with and without the halter placed around the head (6 images in total). All photographs were obtained by the same investigator one day before the gastroscopy examination (see below). After image acquisition, all horses had a complete physical examination performed and their weights obtained either via a weight scale or using a weight tape for approximate weight estimation. 

### 2.3. Lameness Assessment

After photographs for HGS scoring were taken, three consecutive trot ups, in-hand, in a straight line were performed and video recorded for lameness analysis. Subjective lameness assessment was performed independently by the two experienced clinicians (ODS and CS) who were blinded to HGS scores and EGUS grading. A modified American Association of Equine Practitioners (AAEP) lameness scale was used for a straight-line lameness assessment (Table 1). If a discrepancy between lameness scoring occurred, a consensus grading was obtained. Based on the subjective lameness assessment, horses were classified as either being lame (yes) or not lame (no). Further classification included grouping of lameness scores as: 0 = not lame; 1–2 = mildly; 3 = moderately and 4–5 = severely lame. Horses with lameness scores ≥ 2 were excluded from analysis.

### 2.4. Serum Amyloid A Testing (SAA)

Serum amyloid A was used as an exclusion criterion as a concurrent systemic inflammation or an infection of an unknown origin might interfere with HGS. Serum amyloid A measurements were performed as previously described with the use of a validated point of care, stall-side, handheld device (StableLab^®,^, Zoetis, Rhodes, NSW, Australia) [27,28]. Blood collection for SAA measurement was performed after HGS photographs were taken. Horses with SAA values ≥ 50 µg/mL were excluded from final analysis based on previous research and manufacturer recommendations [29].

### 2.5. Gastroscopy Examination and Equine Gastric Ulcer Syndrome Group Allocation

Prior to gastroscopy, all horses were starved for a minimum of 12 h and water was withheld for 2 h. Horses were sedated with 0.01 mg/kg bodyweight detomidine intravenously. The gastroscopy examination was performed using a 300 cm flexible video gastroscope (Aohua VET9830 Video-endoscope, AUSVET Endoscopy, Mt Waverly, VIC, Australia). The gastroscope was passed through a preplaced tube inserted into the cranial esophagus via one nostril. The stomach was insufflated with air and the squamous and glandular mucosa flushed with water to remove any food material that had adhered to the stomach wall. If food remaining in the stomach was too extensive to allow adequate observation of the squamous and glandular mucosa, the gastroscopy examination was repeated after an extended period of starvation. Horses were either starved for another 4–6 h if the gastroscopy was repeated on the same day or starved for 16–20 h if this took place on the following day. Video-recordings were made of all gastroscopy examinations and de-identified to allow for blinded, randomized review by two of the investigators (SF and GFA).

The gastroscopy examination included observation of the squamous fundus, the greater and the lesser curvature of the squamous mucosa, the fundus of the glandular mucosa, the pyloric antrum and pylorus. All regions were graded separately according to the standard Equine Gastric Ulcer Council 5-point ordinal grading system (Table 2). 

Horses were divided into groups based on the presence or absence of overall EGUS, as well as the presence or absence of ESGD or EGGD. Horses were considered to have EGUS if they had an ESGD grade ≥ 2 and/or an EGGD grade ≥ 1. The cut-off points for EGUS clinical significance are poorly defined and criteria for determination of which horses were considered to have EGUS was based on previous research as well as the authors’ experience and clinical decision routinely employed in practice [31,32]. Overall EGUS severity as well as ESGD and EGGD lesion severity were further classified into 3 groups based on their summed grade scale (0 = controls with no disease; 1 and 2 = mild disease; 3 and 4 = moderate to severe disease).

### 2.6. Horse Grimace Scale Scoring

Three veterinarians previously trained and experienced in HGS evaluation and four board certified specialists, without previous HGS scoring experience, performed the HGS scoring. This was performed as previously described [21]. Briefly, a total of six facial action units (FAU) were observed for the presence of ears held stiffly backwards, orbital tightening, tension above eye area, prominent strained chewing muscles, mouth strained and pronounced chin, strained nostrils and flattening of profile. Each FAU from the two lateral and one frontal facial views was graded as either 0 = not present (not seen in any of the three views), 1 = moderately present (based on extent of changes and if seen in one or two views) or 2 = obviously present (based on extent of changes and if seen in all three views). Written instructions on how to grade each facial action unit as well as a total of the six de-identified photographs from each horse were provided to the evaluators. All HGS grading was performed blindly to EGUS grading results and was conducted independently. Median scores were used for each facial action unit (minimum score of 0 and maximum of 2) as well as for an overall HGS (minimum score of 0 and maximum of 12) which was obtained by a summation of all the facial action units results. 

### 2.7. Statistical Analysis

#### 2.7.1. Sample Size Calculation

Based on a standard deviation of 1.5 points on a HGS score in horses with laminitis, a minimum of 17 horses were required in each treatment group in order to avoid a type II error (i.e., a false negative result) [25]. Power analysis was performed with the use of a statistical software IBM SPSS Statistics for Windows, Version 20.0 (IBM Corp, Armonk, NY, USA) using 80% power and an alpha of 5%.

#### 2.7.2. Horse Grimace Scale Inter-Observer Reliability Scores

A two-way mixed intra-class correlation coefficient (ICC) model with 95% confidence interval (CI) was used to determine the level of agreement of HGS scoring between seven observers. Absolute agreement in the rating was chosen to characterize good inter-rater reliability. An intra-class correlation coefficient was calculated for overall HGS and for each FAU across all seven observers, as well as between experienced versus non-experienced observers. Intra-class correlation coefficient values were interpreted as previously described [33]. Briefly, ICC values of 0.75 to 1.0 were considered as “excellent”, 0.60 to 0.74 as “good”, 0.40 to 0.59 as “fair” and ≤0.40 as “poor”. The analyses were performed using the statistical software IBM SPSS Statistics for Windows, Version 20.0 (IBM Corp, New York, NY, USA).

#### 2.7.3. Horse Grimace Scale Scores between Groups

Data distribution was visually assessed via Q-Q plots as well as with D’Agostino–Pearson normality tests. To select for potential confounding factors, univariable linear regression analysis was performed, investigating association of age, sex, breed, weight or SAA with HGS scores. One-way analysis of the variance Welch test was used to assess differences in HGS scores between horses with and without EGUS, ESGD and EGGD. The Brown–Forsythe test was used to determine the difference in HGS results between horses with different severities of EGUS, ESGD and EGGD. The above analyses were performed using the statistical software package R studio (RStudio 4.2.2, Inc., Boston, MA, USA) and GraphPad Prism 9 (GraphPad Prism 6, GraphPad Software Inc., San Diego, CA, USA) with significance set at a *p* ≤ 0.05.

## 3. Results

A total of 77 horses of mixed breeds and age were initially recruited for this study. After the exclusion of horses with concurrent lameness and high SAA values, 61 horses were included in the final analysis. Descriptive statistics of horses are described in Table 3. 

### 3.1. Horse Grimace Scale Inter-Observer Reliability

The inter-observer reliability between all seven observers for overall HGS scores was excellent with an ICC mean (95% CI) of 0.75 (0.65–0.82; *p* < 0.001). Ears stiffly backwards provided the best inter-observer reliability with an ICC value of 0.82 (0.75–0.88; *p* < 0.001), which was followed by strained nostrils (0.73, 0.63–0.81; *p* < 0.001), orbital tightening (0.71; 0.60–0.80; *p* < 0.001) and prominent chewing muscles (0.65, 0.52–0.76; *p* < 0.001). The FAU tension above the eye area and mouth strained with pronounced chin showed fair and poor inter-observer reliability with ICC values of 0.59 (0.43–0.71; *p* < 0.001) and 0.20 (0.07–0.43; *p* < 0.072), respectively. When comparing ICC values between the four specialist veterinarians and three veterinarians experienced in HGS scoring, reliability for overall HGS and for the FAU ears stiffly backwards was similar. However, there were some differences between groups for individual FAU (Table 4). Observers with previous HGS scoring experience demonstrated good orbital tightening ICC scores (0.68) compared to fair ICC (0.58) for veterinary specialists. In contrast, previously HGS trained observers demonstrated poor ICC scores for tension above the eye (0.26) and prominent and strained chewing muscles (0.24) compared to veterinary specialists with ICC scores of 0.52 and 0.66, respectively.

### 3.2. Horse Grimace Scale Scores between Horses with and without EGUS

**Figure 1:**
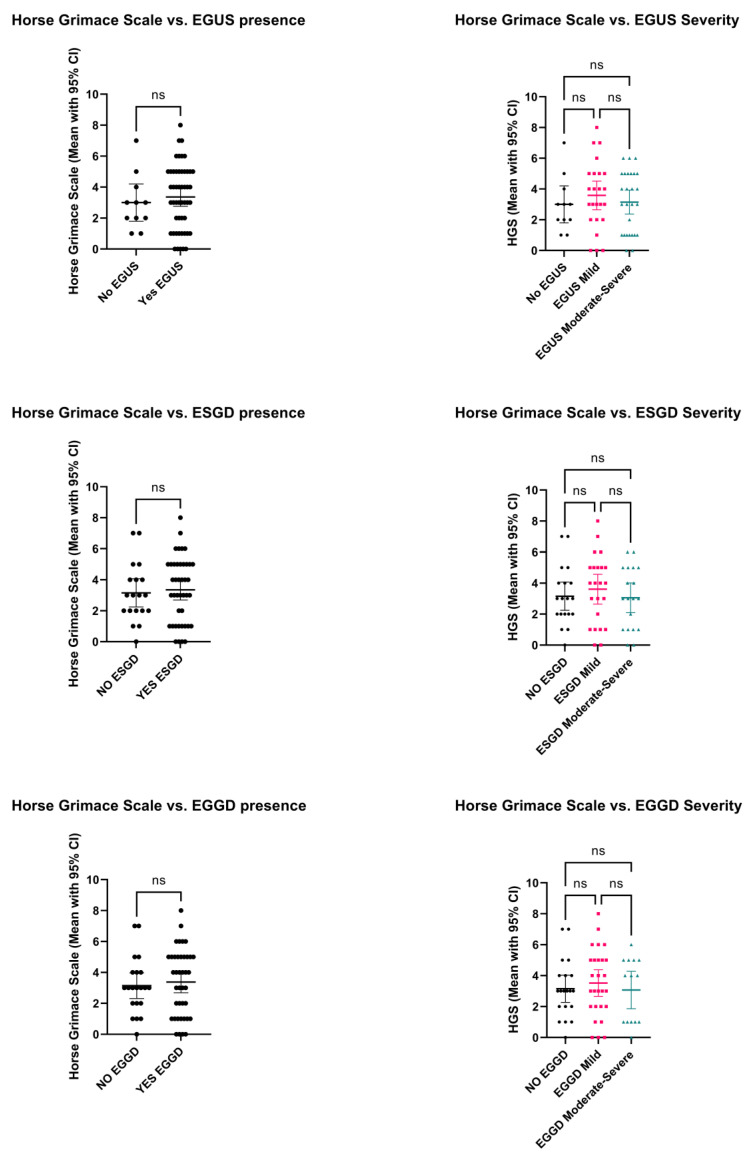
Horse grimace scale scores (mean, 95% CI) between horses with and without EGUS, ESGD and EGGD (plots on the left). Differences of HGS scores (mean, 95% CI) between control horses (NO—dots) and the different severities groups (mild [squares] and moderate to severe [triangles]) for EGUS, ESGD and EGGD are demonstrated on the plots on the right. Differences between all groups were not significant (ns: *p* > 0.05).

The median HGS scores obtained from all seven observers were used for comparisons between horses with (*n* = 50) and without EGUS (*n* = 11), ESGD (*n* = 42) and EGGD (*n* = 40) as well as HGS related to EGUS severity which are depicted in Figure 1. No effect of age, breed, sex, weight or SAA on HGS scores were observed. No differences were observed on overall HGS scores in horses with EGUS (mean, 95% CI; 3.36, 2.76–3.95) compared to horses without EGUS (3, 1.79–4.20), *p* = 0.566. Similarly, no effect was found when comparing HGS between horses with (3.35, 2.69–4.01) and without ESGD (3.15, 2.24–4.07) as well as with (3.37, 2.68–4.06) and without EGGD (3.14, 2.31–3.97) with a *p* value of 0.715 and 0.659, respectively. No significant differences (*p* = 0.656) in HGS were found between different severities of gastric lesions with horses with mild or moderate to severe EGSD and EGGD showing similar HGS scores to control horses. 

## 4. Discussion

The study presented herein investigated the use of HGS in horses with and without EGUS and whether observer experience in interpreting HGS influenced the results. The hypothesis that HGS scores would be higher in horses suffering from EGUS was not proven. Furthermore, HGS inter-observer reliability was not improved by previous HGS scoring experience.

### 4.1. The Use of a Pain Scale for Investigating Pain in Horses with EGUS

The investigation of pain levels associated with different disease processes is important to help establish their impact on animal welfare. Knowledge regarding assessment of painful behaviour via different pain scales is becoming more readily available for use in both research and in the clinical setting. Additionally, the choice of which pain scale should be implemented is also important. A recent meta-analysis evaluating facial expression of pain in nonhuman mammals concluded that the HGS had an overall high level of evidence for its measurement properties [34]. Due to the practicality and easy implementation of the HGS in the field and in the clinic setting, this measure was deemed an appropriate choice for an initial investigation of pain in horses with gastric ulcers. 

### 4.2. Horse Grimace Scale Scores in Horses with and without Gastric Ulcers

In this current study, there were no differences in HGS between groups. A study using the HGS to assess pain in horses with acute laminitis before and after treatment reported HGS scores with a mean ± SD of 5.0 ± 2.6 before treatment (painful horses) and 3.5 ± 2.3 after treatment (non-painful horses) [25]. Quantification of pain in horses with dental disorders revealed median HGS of 4 in horses with dental disease [27]. In our study, mean HGS scores of 3.36 and 3 were obtained in horses with and without EGUS, respectively. Comparing the HGS in the laminitis and dental studies with the HGS of horses obtained in our study, aside from a few outliers, most of the horses in this present study had a non-painful HGS score, regardless of group. 

When comparing HGS with the presence or not of EGUS in our study, only 11 horses had no evidence of gastric lesions versus 50 horses with gastric lesions, which could have caused a sample size effect and contributed to the lack of statistical significance. Similarly, when comparing the HGS scores between the different severity groups, gastric lesions graded as 3 and 4 were allocated within the severe group in order to provide strength for sample size within this group. It is possible that, if more horses with grade 4 squamous or glandular lesions were included, differences between severely affected animals and controls could have been observed. 

Equine gastric ulcer syndrome is more likely to be considered a chronic pathological condition although it can occur with an acute onset. It is possible that the HGS may not be sensitive enough to detect chronic pain states. The HGS was initially studied in association with acute pain, such as castration and laminitis [21,25]. One study successfully identified chronic pain in horses with long term dental disorders using the HGS [26]. It is possible that a chronic pain state originating from around the face could increase the HGS score more than a chronic pain related to visceral pain such as EGUS. Further studies investigating the use of HGS to assess other causes of chronic pain are required to establish this assumption. 

It is also possible that pain related to EGUS may only occur intermittently, rather than being continuously present. In humans, an increase in pain levels occurs when peptic ulcers are exposed to hydrochloric acid [14]. It is conceivable that in horses, pain might be experienced only when gastric pH levels are lowest or when acid splashes against damaged squamous mucosa, such as during exercise [1,35,36,37]. Further research investigating pain assessment in horses with and without EGUS with the use of the HGS and other pain scales before and after exercise is warranted. 

### 4.3. Horse Grimace Scale Inter-Observer Reliability

The overall HGS inter-observer reliability found in this study is similar to, but lower than, that observed in previous studies using HGS for different painful conditions [21,25,26,38,39]. The HGS scoring investigated in horses before and after routine castration showed excellent inter-observer reliability with an ICC value of 0.92 [21]. Whereas the ICC, demonstrated in our study, although categorized as “excellent”, was of a lower value (0.75). When comparing the inter-observer reliability for each FAU, ears stiffly backwards had excellent ICC scores in our study (0.89), similar to previous HGS publications evaluating HGS in chronic dental disorders and after castration [21,26]. Likewise, HGS assessment in horses with acute laminitis showed very good ICC for most but not all FAUs [25]. In accordance with the recent literature, FAUs such as prominent strained chewing muscles, strained nostrils and flattening of the profile, orbital tightening and mouth strained and pronounced chin had the lowest ICC scores [25,38]. 

Face-to-face training sessions can improve the inter-observer reliability of some (stiffly backwards ears, orbital tightening and prominent strained chewing muscles) but not all FAU scores [39]. Direct face-to-face HGS training sessions were not provided in our study and could have been conducted prior to grading in order to improve inter-observer reliability. Unfortunately, the training session material used in the research aforementioned is not available in the public domain and, for this reason, was not implemented in our study. In an attempt to standardize HGS scoring between observers, instructions on how to use the HGS, based on previous studies of HGS, were provided to all observers prior to any grading [21].

Subjective rating scales can be vulnerable to bias and challenges during grading. Some FAUs can be more difficult to assess due to interference with halter placement and positioning [26]. Therefore, images with and without the halter around the horse’s face were provided in order to aid observation of all FAUs appropriately. In an attempt to decrease bias, we recruited a total of seven observers who were completely blind to horse general health, SAA results, presence of lameness and EGUS results. All observers were experienced veterinarians, four of whom had extensive experience as veterinary specialists in different areas with routine practice in examining horses suffering from different painful conditions. In addition, three observers had no previous knowledge in HGS scoring in painful and non-painful conditions. In contrast to a previous investigation, HGS scoring experience did not improve overall ICC values in our study [39]. However, there were differences for individual FAUs. It is possible that some FAUs such as tension above the eye and prominent and strained chewing muscles, which had better ICC scores from the veterinary specialists, might be more commonly used in clinical practice and would account for this difference. Therefore, extensive clinical experience might improve inter-observer reliability. The difference in ICC scores seen is also in line with previous studies where specific FAUs were more difficult to grade compared to others which can lead to an impact in ICC values [21]. It is noteworthy that most of the HGS-related research has been published by the same research group who developed and validated this pain scale. The vast experience in HGS grading from these researchers may be related to the higher inter-observer reliability scores observed in previous publications [21,25,38,39,40]. A standardized training protocol for improvement of HGS inter-observer reliability as well as further HGS studies from different research groups evaluating HGS scores reliability might be of value. 

### 4.4. Study Limitations

A possible reason for the lack of differences in HGS between groups is the methodology used. Previous studies using the HGS used more controlled environments such as housing horses in enclosed stables for at least an hour before assessment and taking still images from videos to avoid any variables such as environmental factors or the presence of people which could change a horse’s facial expression [21]. However, no evidence of variation in overall HGS scores was noted in one study when horses were exposed to different stimulants such as a new environment, grooming, food or a frightening event [38]. Whilst the study found that the overall HGS was not significantly different between the different stimuli, increased scores for specific FAU such as “ears stiffly backward” and “prominent strained chewing muscles” occurred when the horses were frightened [38]. As horses in this study had HGS photographs taken in various situations that could cause fear or anxiety, such as a windy day or the introduction to a new environment, the scores could be increased enough to affect results. Further investigations should aim to control these extraneous variables which was not feasible in our study as horses were evaluated in a real-life setting, such as in training facilities and on farm. For practicality and time purposes, we acquired images for HGS after a 5 min acclimatization period (when horses were evaluated in their own environment—on farm) so horses could get used to the handler and a 20 min period of acclimatization to the environment with additional 5 min acclimatization to the handler when horses were evaluated at the University of Adelaide facilities. 

The use of still images taken from serial photographs rather than still images from video-recording might have been a limitation. Specifically, the facial action units of orbital tightening and ears stiffly backwards could have been erroneously graded if the image was acquired at the same time as blinking or when the ears were moving due to background noise [21,25,26]. Furthermore, in order to attenuate any effect of using photographs rather than still images randomly captured from video recordings, serial photographs were taken from each horse, with the photographer not facing the horse and in a as neutral position as possible, as previously reported [26]. All photographs were carefully taken when horses were not distracted by the environment or the handler. Previous studies investigating the use of HGS to identify and quantify pain due to dental disorders in horses also used still images with no effect of photographic imaging on the ability of using HGS to identify pain [26]. 

Finally, another limitation in our study was the potential for other types of pain being present within the cohort studied and acting as a cofounding factor. In order to decrease the impact of other painful conditions, horses with lameness or significant inflammation were excluded from the data analysis. All horses included in our study were reported to be up to date with general veterinary care which included routine dental health evaluations. However, given that an oral examination was not performed, it is possible that concurrent dental disease or bit-related oral lesions could have had an impact on HGS scores. 

## 5. Conclusions

Equine gastric ulcer syndrome is extremely prevalent and understanding the impact of this disease on pain and horse welfare is of importance. The hypothesis that horses with EGUS would have higher HGS scores than their healthy counterparts was not supported by the results of this study. Further studies with increased numbers and alternative measurements of pain are required to draw firm conclusions regarding the influence of EGUS on horse welfare.

## Figures and Tables

**Table 1:** Modified AAEP lameness scale for straight line lameness assessment.

Grade	Severity
0	No lameness is visible.
0.5	Lameness difficult to observe or is not consistent and repeatable.
1	Forelimb lameness involves a discernible head nod; however, full symmetrical limb movement is present. Hindlimb lameness involves a mild hip drop or hip hike only.
2	Forelimb lameness involves a consistent and moderate head nod and asymmetrical limb placement. Hindlimb lameness involves a discernible and consistent hip drop and hip hike.
3	Forelimb lameness is consistent and marked; however, lameness is not visible at the walk. Limb placement may be obviously altered and ground contact may not be complete in the affected limb. Hindlimb lameness involves an obvious hip drop and hip hike and may result in a compensatory head nod.
4	Forelimb and/or hindlimb lameness is visible at the walk.
5	Lameness produces minimal weight bearing in motion or at rest or a complete inability to move.

**Table 2:** Grading system for Equine Squamous and Glandular Gastric Disease as recommended by the Equine Gastric Ulceration Council 5-point ordinal grading [30].

Grade	Squamous Mucosa	Glandular Mucosa
0	The epithelium is intact and there is no appearance of hyperkeratosis	The epithelium is intact and there is no obvious hyperemia
1	The mucosa is intact, but there are areas of hyperkeratosis	The epithelium is intact and there are areas of hyperemia
2	Small, single or multifocal lesions	Small, single or multifocal lesions
3	Large single or extensive superficial lesions	Large single or extensive superficial lesions
4	Extensive lesions with areas of apparent deep ulceration	Extensive lesions with areas of apparent deep ulceration

**Table 3:** Descriptive data for 61 horses initially recruited into this study. Horses described according to breed, gender, age and weight (mean (min–max)).

Breed	Gender	Age (Years)	Weight (kg)
Appaloosa 1 Arabian 2 Brumby 1 Clydesdale 1 Riding pony 4 Standardbred 39 Thoroughbred 8 Warmblood 3 Unknown 2	Geldings 29 Mares 32	8.3 ± (2–18)	476.6 (296–595)

**Table 4:** Inter-observer reliability scores measured by ICC between different observers for overall HGS as well as across six FAU (mean, 95% CI).

ICC *	All Observers (*n* = 7)	Veterinary Specialists (*n* = 4)	Veterinarians with HGS Experience (*n* = 3)
Overall HGS	0.75 (0.65–0.82)	0.64 (0.49–0.75)	0.61 (0.43–0.74)
Ears stiffly backwards	0.89 (0.85–0.92)	0.82 (0.75–0.88)	0.84 (0.75–0.89)
Orbital tightening	0.71 (0.60–0.80)	0.58 (0.40–0.72)	0.68 (0.54–0.79)
Tension above the eye	0.59 (0.43–0.71)	0.52 (0.33–0.67)	0.26 (0.05–0.49)
Prominent and strained chewing muscles	0.65 (0.52–0.76)	0.66 (0.52–0.77)	0.24 (0.09–0.48)
Mouth strained and pronounced chin	0.20 (0.07–0.43)	0.23 (0.06–0.46)	0.12 (0.23–0.39)
Strained nostrils and flattening of profile	0.73 (0.63–0.81)	0.60 (0.44–0.73)	0.59 (0.40–0.72)

* ICC interpretation: “excellent” (0.75–1.0), “good” (0.60–0.74), “fair” (0.4–0.59), “poor” (0–0.40) [33].

## Data Availability

Not applicable.
